# Mechanisms of human color constancy underpinning stable visual perception

**DOI:** 10.11470/jsaprev.240213

**Published:** 2024-11-06

**Authors:** Takuma Morimoto

**Affiliations:** Department of Experimental Psychology, University of Oxford, Oxford OX2 6NW, United Kingdom

## Abstract

When we refer to “a green car,” it might sound as if that the color is an inherent characteristic of the object. However, color is a sensation produced by our visual system based on the light reflected from an object. Changes in scene illuminant alter the reflected light, so the color of the object should change as well. Yet, why does a green car parked in the morning still appear green when we return to it at dusk? This is due to color constancy, a phenomenon that maintains stable color perception despite changes in surrounding lighting environments. How does our visual system create a robust visual world from highly variable sensory signals? This article explains the mechanisms supporting human color constancy.

## 1 Introduction

**Fig. 1:**
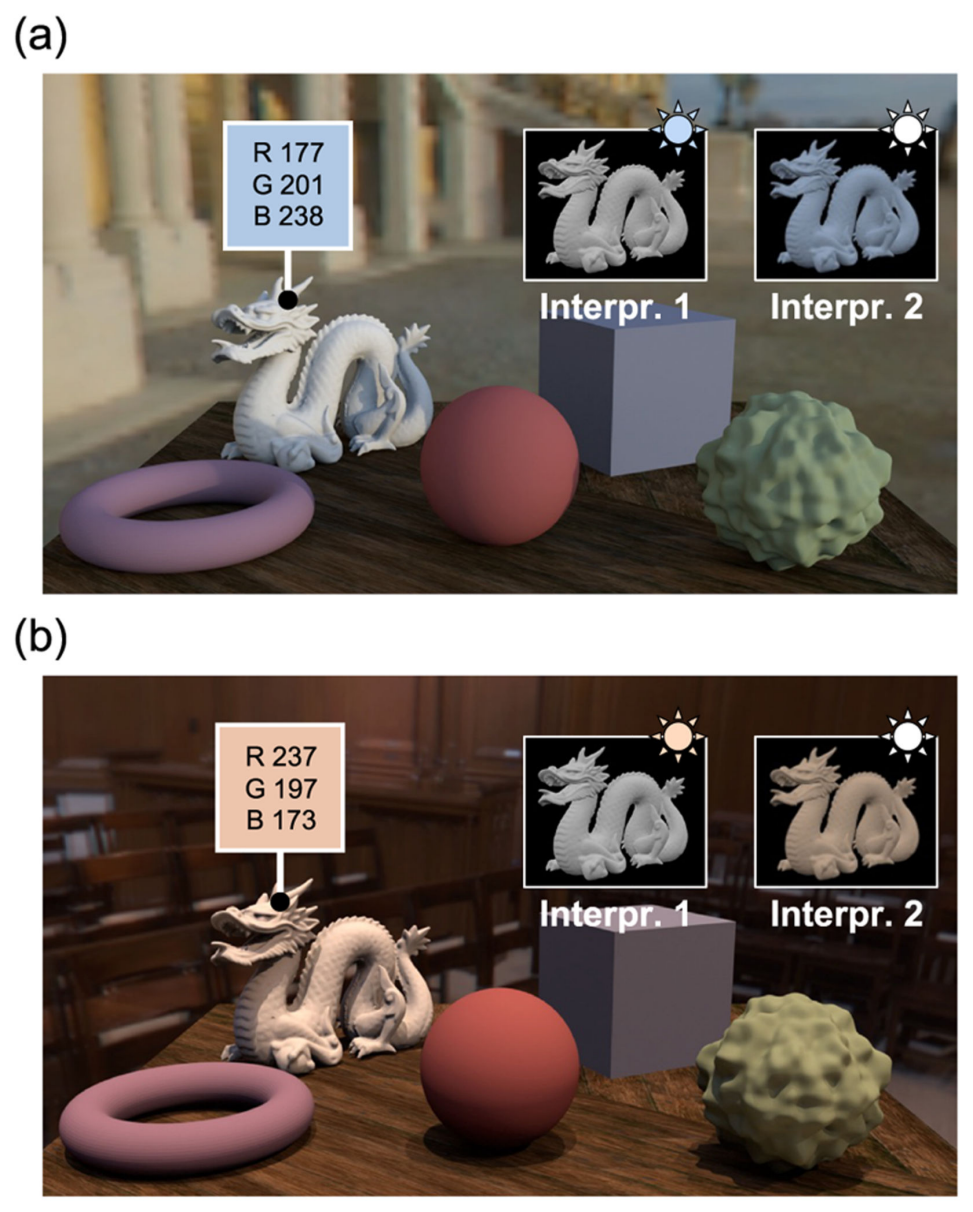
(a), (b) The same group of objects under different lighting. Although the pixel values on the forehead of the white dragon differ greatly due to differences in the surrounding lighting environment, we can still recognize them as white objects. How do we achieve color constancy despite multiple interpretations of the combination of lighting color and object color?

Color is a concept we commonly use to identify objects (e.g. could you pass me the blue coat?). However, objects themselves have no inherent color. Instead, color is a sensation that occurs when light from a scene’s illuminant reflects off an object’s surface and enters our eyes. When the illuminant changes, the reflected light also changes, suggesting that the color of the object should change; however, this is not what we perceive. Figures 1(a) and 1(b) display the same group of objects under different lighting conditions. The dragon figurine appears white in both settings. However, checking the color of a single pixel in the forehead region reveals significant differences due to the surrounding lighting environment. This stable color perception is possible because of color constancy, which compensates for the lighting environment. To achieve this, one might consider subtracting the illuminant components from the reflected light entering the eye. However, how do we determine whether the dragon figurine is a white dragon under cool/warm lighting (interpretation 1) or a cool/warm colored dragon under white lighting (interpretation 2), as illustrated in the upper right corner of the scene?

This article explores the intriguing phenomenon of color constancy from four perspectives. First, it examines how the amount of response of the three types of photoreceptor cells (cones) in the retina changes with different lighting conditions. Next, it introduces the statistical characteristics of light and objects in natural environments, identified through physical measurements, that our visual system can utilize. Third, it explains how cone responses for each surface change with lighting when considering natural illuminants or object surfaces, and introduces a signal-transformation function to adjust cone responses to undo changes in illuminants. Finally, it presents visual cues for estimating the illuminant color, used to adjust cone responses, as proposed in previous studies.

Although color constancy is a mechanism of the sensory system, understanding it requires comprehensive investigation of the physical aspects of light and objects that provide input to our visual system. Thus, color constancy is closely tied to physics, and the related findings have broad applications. For example, white-balance technology, which adjusts the color tone of images captured under different lighting, is fundamental for image color correction. In computer vision, particularly in developing robotic vision systems, this technology contributes to stable operation at night, in backlit environments, and during low-visibility conditions such as rain.

## 2 Challenges underlying color constancy

**Fig. 2:**
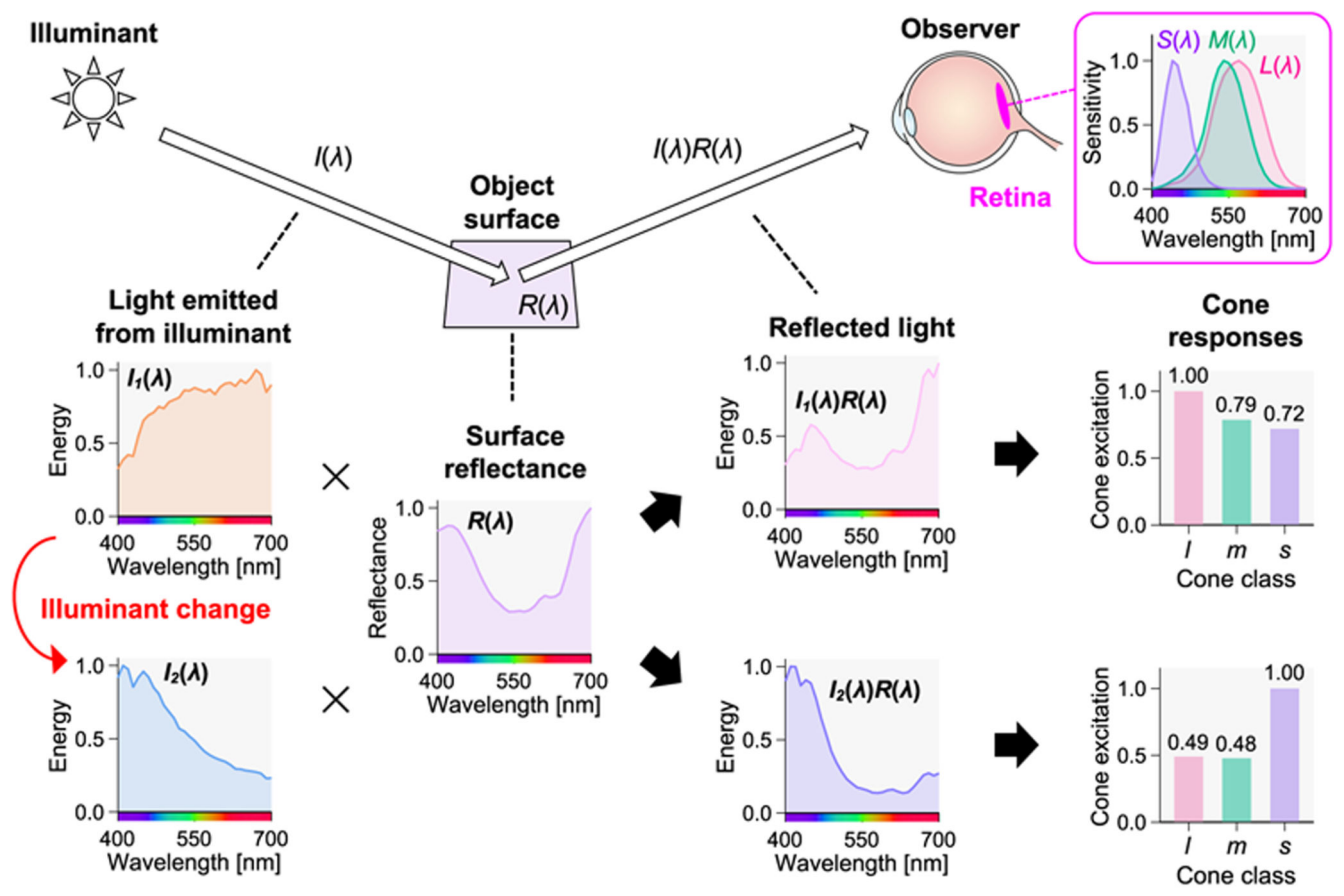
The effect of changes in illumination on the reflected light from objects and the amount of cone responses. Light *I*_1_(*λ*) emitted from the illumination is reflected on the surface of an object with a reflectance *R*(*λ*), and the reflected light *I*_1_(*λ*) *R*(*λ*) enters the observer's eye. The light is then absorbed by cone photoreceptors on the retina, producing LMS cone responses. When the illumination changes to *I*_2_(*λ*), the reflected light also changes to *I*_2_(*λ*) *R*(*λ*), causing significant changes in the cone signals. The problem of color constancy is how to produce the same color from cone signals that have changed significantly due to lighting.

To understand the challenges in color constancy, we consider the types of problems faced by our visual system. In Fig. 2, a person observes an object surface under a specific illuminant condition. On the left side of the figure, the spectral energy (intensity for each wavelength) *I*_1_(*λ*) of the light emitted from the illuminant contains high energy on the long-wavelength region, appearing orange when viewed directly. The object has a spectral reflectance *R*(*λ*), which represents the proportion of energy reflected by the object at each wavelength. In this example, the surface effectively reflects the long- and short-wavelength lights and appears purple under a white illuminant. The incident light *I*_1_(*λ*) is reflected by the object with spectral reflectance *R*(*λ*), and the reflected light *I*_1_(*λ*)*R*(*λ*) enters the observer’s eye. The retina at the back of the eye contains three types of photoreceptor cells that respond to light: long-wavelength-sensitive (L), middle-wavelength-sensitive (M), and short-wavelength-sensitive (S) cones. The sensitivity of the cones for each wavelength (the spectral sensitivity function) has been experimentally identified [1]. By multiplying each of *L*(*λ*) , *M*(*λ*), and *S*(*λ*) by the spectral distribution of light and then integrating over the wavelength, the responses *l, m*, and *s* of each cone to this light can be obtained using Eqs. (1), (2), and (3), respectively.

(1) $$l=\int L(\lambda )I_{1}(\lambda )R(\lambda )d\lambda$$

(2) $$m=\int M(\lambda )I_{1}(\lambda )R(\lambda )d\lambda$$

(3) $$s=\int S(\lambda )I_{1}(\lambda )R(\lambda )d\lambda$$

Under the orange light in Fig. 2, the responses of the L-, M-, and S-cones to the reflected light *I*_1_(*λ*)*R*(*λ*) are 1.00, 0.79, and 0.72, respectively, and these three values form the basis of color perception. Next, consider the scenario where the illuminant changes to cool light *I*_2_(*λ*) with high energy on the short-wavelength region. The reflectance of the object remains *R*(*λ*) ; however, the reflected light changes to *I*_2_(*λ*)*R*(*λ*) with the illuminant change, and the responses of the L-, M-, and S-cones change significantly to 0.49, 0.48, and 1.00, respectively. The question to be addressed is as follows: Why can we perceive the color of an object’s surface consistently even though the cone signals at the entrance of the visual system change significantly with the illuminant change? Notably, countless combinations of lighting and surfaces can yield the same responses from L-, M-, and S-cones. Thus, computing the illuminant color from the cone response to one surface is an ill-posed problem, making the color constancy problem challenging to solve.

## 3 Statistical characteristics of light and objects in natural world

Color constancy is challenging to solve. However, it is important to consider whether the physical properties of lighting and objects in the real world exhibit helpful characteristics or regularity. If these can be identified and used as prior knowledge, the visual system may not need to seek the answer from countless combinations. Considering the evolution of the visual system, the mechanisms of color constancy may have developed to utilize the statistical regularities of natural light and natural objects that are particularly relevant to us [2,3].

**Fig. 3:**
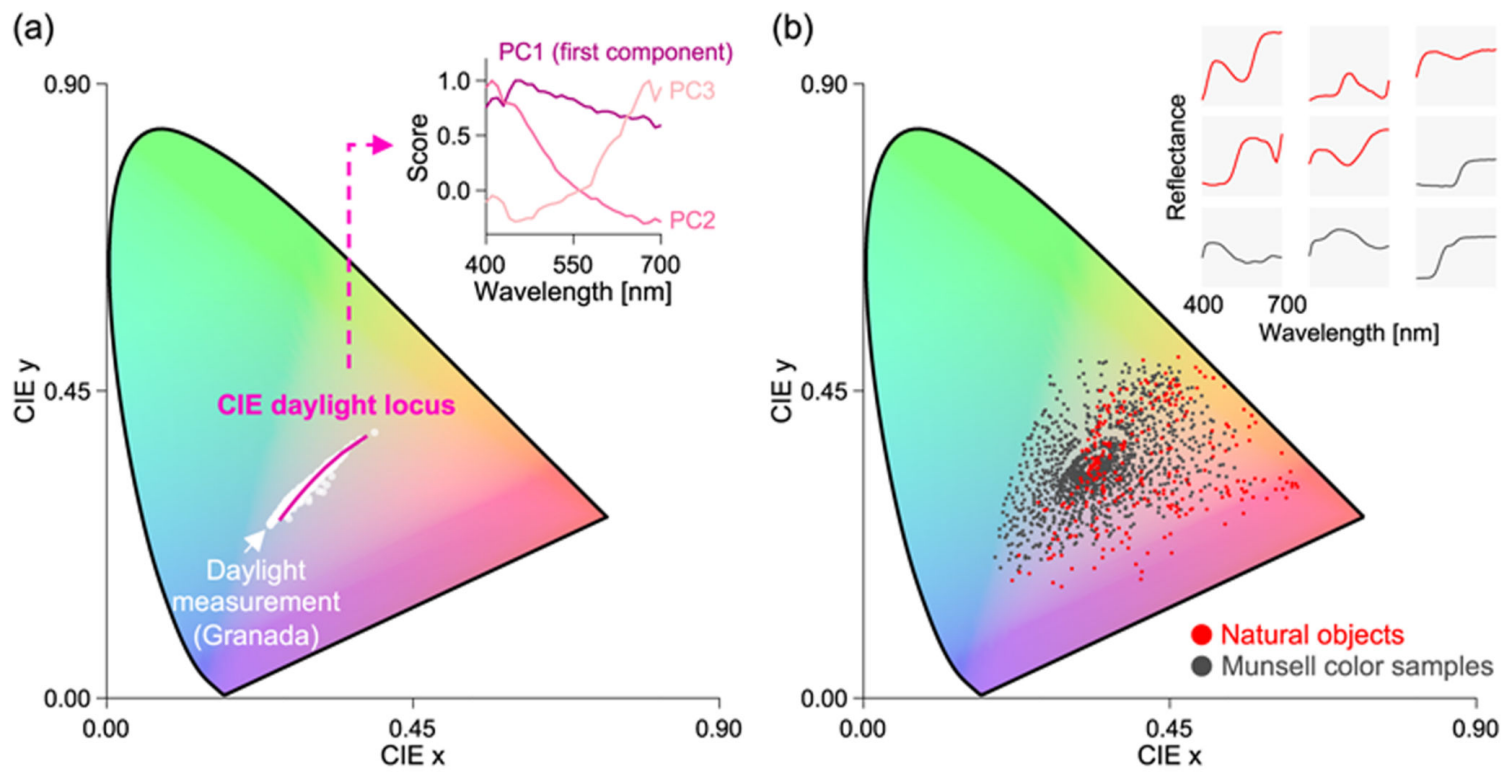
Real-world illuminant and object color distribution. (a) The daylight locus drawn by the measured color distribution of natural light and the basis functions of wavelength *λ* obtained through principal component analysis. The white dots represent the results of 2600 natural daylight measurements in southern Spain. (b) The color distribution of natural objects (red dots) and Munsell color samples (black dots). To show examples of the shapes of spectral reflectance, the top right displays the spectral reflectance of five natural objects (red lines) and four Munsell color samples (black lines).

The analysis of 622 daylight spectra measured in the UK, US, and Canada is widely known [4]. Principal component analysis revealed that the spectral distribution of natural illuminant can be accurately represented by just three smooth basis functions of wavelength *λ*, despite natural spectral fluctuations due to time of day and weather. Notably, light synthesized using these basis functions have a continuous and smooth spectral energy distribution, a key statistical feature of natural light. Additionally, plotting this synthesized light on a chromaticity diagram, which numerically represents colors of light, showed that natural light is distributed in a small region within the wide color space, mainly along the yellow–white–blue axis (Fig. 3(a), the Commission Internationale de l’Eclairage (CIE) daylight locus). One may notice that green and pink lighting are not observed in daily life, except with artificial lighting. Thus, the colors of natural light exhibit high regularity. Furthermore, spectral measurements of 2,600 lights in Southern Spain (Fig. 3(a), white dots) shows the same trend [5]. This observation may hold true across different countries.

Similarly, the reflectance of natural objects such as flowers, leaves, vegetables, fruits, and tree trunks has been measured [6–11]. The chromaticities of 307 objects [11] calculated under a white illuminant are depicted as red dots inFig. 3(b). They are organized in a relatively narrow region of the color space, though less narrow than the distribution of natural illuminant. Many natural objects are orange, yellow, yellow–green, and green, whereas blue–green or highly saturated green objects are absent here. The black dots in the figure show the internationally standardized color sample of 1,269 Munsell colors [12]. Developed as a color sample, it includes a wide range of colors, but its distribution is only slightly broader than that of natural objects. This is partly because it is limited by the types of paints and inks available, making it difficult to create colors with extremely high saturation levels. The top right of Fig. 3(b) shows examples of the spectral reflectance of natural objects (red line) and Munsell color samples (black line). Therefore, natural objects and even artificial Munsell color samples tend to have smooth spectral reflectance functions, implying that objects in the real world exhibit statistical features similar to those of natural light.

Statistical models have been proposed to estimate lighting and objects’ reflectance using this typical range of colors and the smoothness of the spectral distribution as prior knowledge, leading to high estimation accuracy [13,14]. Additionally, reevaluating the color constancy problem by focusing on natural objects and natural illuminants has revealed some important findings, which are described in the next section.

## 4 Transformation of cone responses to discount illuminant changes

Statistical regularities exist in the physical characteristics of natural light and objects. Thus, one may ask: When lighting changes in the external world, do the cone signals also change in a specific manner? What type of signal conversion is necessary to undo that change?

**Fig. 4:**
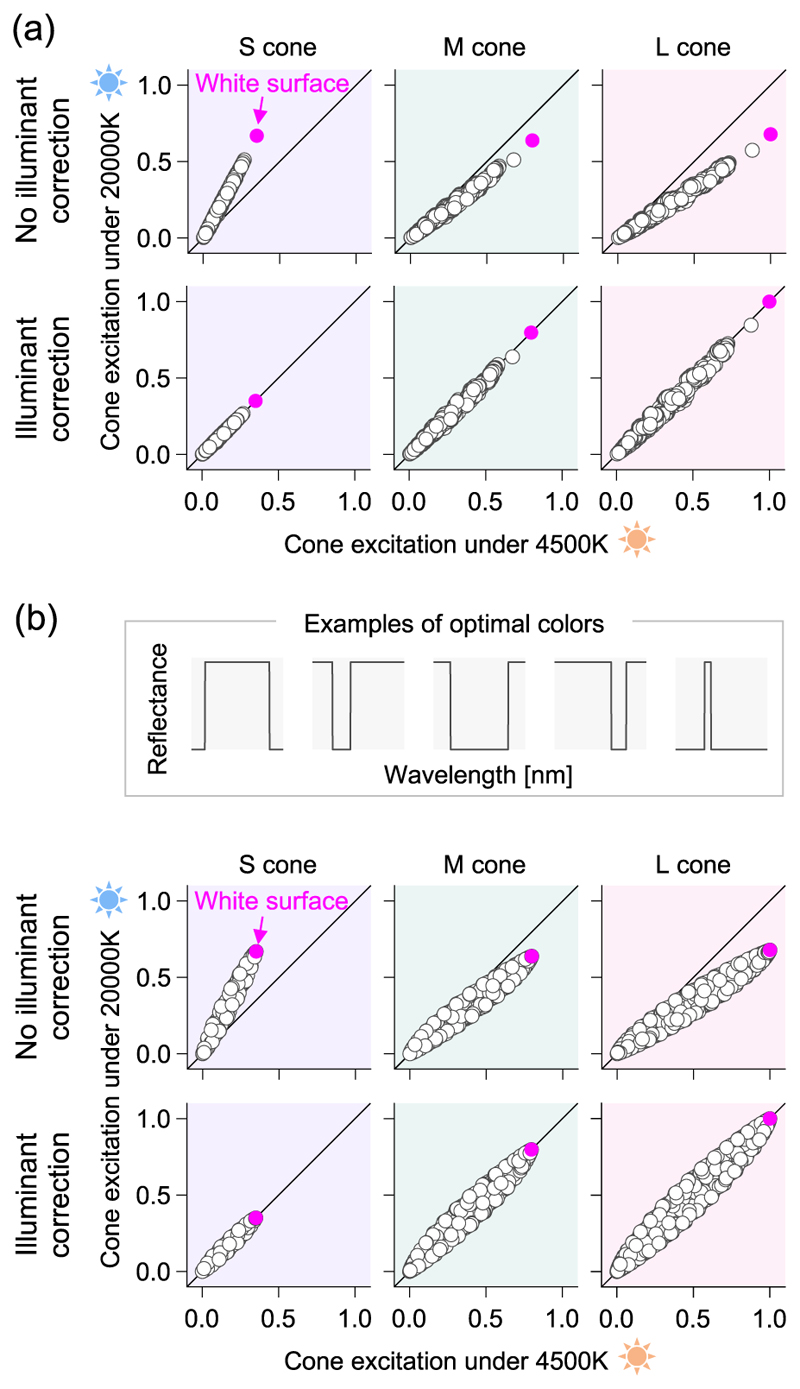
(a) The upper plot shows the relationship of LMS cone responses for 1576 colors, combining natural objects and Munsell color samples under two illuminants (4500K and 20000 K). The lower plot shows the cone response values after correcting for illuminant effects using the Ives transformation, with most data points aligning along the diagonal, indicating color constancy. (b) The relationship of LMS cone response values when using a hypothetical surface, known as the optimal color. The upper part shows examples of spectral reflectance of five optimal colors. Even after the Ives transformation, many data points do not align along the diagonal.

To explore this, assume an illuminant change from a warm natural to a cool natural illuminant, corresponding to color temperatures 4,500K and 20,000K [15], representing the color of light as temperature. The upper part of Fig. 4(a) shows the cone responses for 1576 colors (natural objects and the Munsell color samples) when placed under each illuminant. We see that for all L-, M-, and S-cones, the points aligns closely on a straight line, showing that the cone signals are strongly correlated even when the color of the illuminant changes. This near-perfect correlation arises from the smooth spectral distribution of natural surfaces and illuminants. The tip of the straight line represents a full white surface, reflecting 100% at all wavelengths, which corresponds to the chromaticity of the illuminant itself. This implies that the illuminant change can be effectively canceled by estimating the cone responses of the illuminant under each illuminant condition and multiplying the ratio by the cone responses for all surfaces. This transformation is expressed in Eq. (4) using a diagonal matrix.

(4) $$\left(\begin{array}{c} L_{n}^{\prime }\\ M_{n}^{\prime }\\ S_{\text{n}}^{\prime } \end{array}\right)=\left(\begin{array}{ccc} L_{i1}/L_{i2} & 0 & 0\\ 0 & M_{i1}/M_{i2} & 0\\ 0 & 0 & S_{i1}/S_{i2} \end{array}\right)\left(\begin{array}{c} L_{n}\\ M_{n}\\ S_{n} \end{array}\right)$$

Here, *L*_*n*_, *M*_*n*_, and *S*_*n*_ are the cone responses before the transformation; and $L_{n}^{\prime },M_{n}^{\prime }$, and $S_{n}^{\prime }$ are the cone responses after the transformation (*n* = 1, 2, …, 1576); *L*_*i*1_, *M*_*i*1_, and *S*_*i*1_ are the cone responses of 4,500K illuminant obtained from a full white surface; and *L*_*i*2_, *M*_*i*2_, and *S*_*i*2_ are the cone responses of 20,000K illuminant. This transformation, proposed by Ives in 1912 as a mechanism for color constancy, is known as the “Ives transformation” [16].

The converted cone signals are shown at the bottom of Fig. 4(a). The data points align well on the diagonal line, effectively undoing the sensory signal change caused by the illuminant change. It is important to note that no mathematical basis ensures data points should lie on a straight line across different illuminants, as in this example. Consequently, even if the illuminant color is determined accurately, subtracting the illuminant influence via a simple multiplication as in Eq. (4) may not always be feasible [17]. In fact, when considering a hypothetical surface with extreme reflectance (or the optimal color) [18], points deviate from the line, and many surfaces remain deviated from the diagonal line even after the Ives transformation (Fig. 4(b)). Similar issues arise with illuminants that have extreme spectral distributions. In other words, the statistical features of objects and lights in the real world make color constancy a more tractable problem. Considering that each cone can change its sensitivity through adaptation, the Ives transformation is physiological plausible. However, one question remains: How does the visual system determines the parameters used in the transformation, i.e., the color of the illuminant?

## 5 Visual cues for estimating illuminant color

This section introduces computational methods for our visual system to estimate the illuminant color using cues from the scene.

### 5.1 “Brightest is white” assumption

A completely white surface having a reflectance of 100% at all wavelengths reflects the incident light as it is. This directly aids in illuminant estimation if present. However, a white surface may not exist in a scene; even if it does, one cannot determine whether the surface is white without color constancy. Therefore, the “Brightest is white” heuristic assumes that the brightest surface in a scene is white and can estimate the illuminant color [19]. The accuracy of this assumption depends on the extent to which the brightest surface in a scene resembles white.

### 5.2 “Gray world” assumption and average color

**Fig. 5:**
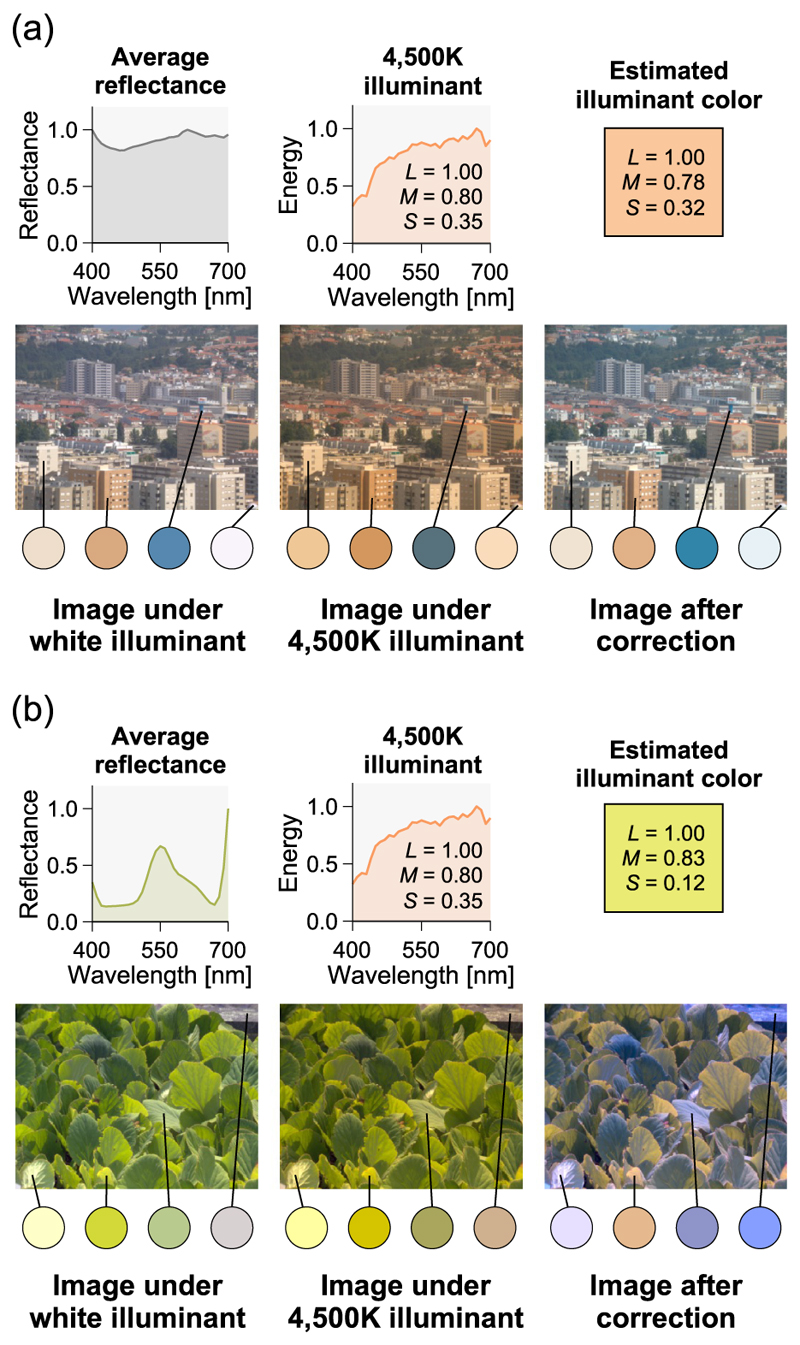
Estimation of illuminant color using the average color of a scene and correction of illuminant using the Ives transformation. (a) An example where the average reflectance of the scene is flat, and the correction is successful. (b) An example where the scene’s object colors are biased, and the correction is not successful. To show the effect of illumination, the four local color changes in the scene are shown below each image.

The color of a scene shifts toward the illuminant color when the scene illuminant changes. If the average reflectance of objects in a scene is neutral (i.e., the “gray world” assumption), deviations from gray indicate the effect of lighting [20]. Figure 5 shows the results of the illuminant correction using the average color (a) in a scene where the “gray world” assumption holds and (b) in a scene where it does not hold [21,22]. Both are simulation of changing the scene illuminant from white (left image) to warm (center image). In the center image, the spatial average color was calculated to estimate the illuminant color as shown in the top right. Then, the Ives transformation was applied to all pixel colors in the center image. In panel (a), the estimated illuminant color (*L* = 1.00, *M* = 0.78, *S* = 0.32) is close to the true illuminant color (*L* = 1.00, *M* = 0.80, *S* = 0.35), and the transformation effectively discounted the illuminant influence. Meanwhile, in panel (b) the scene was biased to green, and the estimated illuminant was clearly drawn toward the color of the object, resulting in a blue-biased corrected image. As demonstrated here, the average color is not highly effective in the scene where the “gray world” assumption does not hold, and the average reflectance is not necessarily flat in actual scenes [23]. However, because of its simplicity, the average color is still regarded as a potential cue for estimating illuminant color.

### 5.3 Correlation between chromaticity and luminance

When a white scene under a red illuminant and a red scene under a white illuminant have the same average color, how does the visual system distinguish between them? This issue is addressed by examining the correlation between chromaticity and luminance [24]. Analyses of natural images with varying illuminant colors revealed that the luminance of red objects increased under a red illuminant, whereas under white lighting no such correlation was observed. This is because the energy of a red illuminant is mainly in the long-wavelength region, and red objects reflect that wavelength region well, resulting in increased luminance. Conversely, a white illuminant has similar energy across wavelengths, showing minimal variation in luminance across objects with different colors. Experimental findings suggest that the visual system uses this correlation as a cue to correctly distinguish the illuminant, even when the average color matches.

### 5.4 Geometry of chromaticity versus luminance distribution

Combinations of chromaticity and luminance of objects placed in a scene exhibit systematic regularity depending on the illuminant color of the scene. Based on this observation, a computational model has been proposed to estimate the illuminant color by analyzing the geometry of chromaticity versus luminance distribution. Behavioural experiments showed that this model effectively predicts the illuminant color estimated by human observers [25,26]. Although this method is challenging in scenes lacking objects with diverse reflectances, it explains individual differences in perception, such as the “#theDress” phenomenon, where the dress appears as either blue and black or white and gold [27].

### 5.5 Memory color

If the color of an object under a white illuminant is known, such as a red tomato, any shift from this color can be attributed to the effect of the illuminant [28]. While simple and effective, this method is not applicable to abstract objects without prior knowledge or when features other than color, such as in the cases of lemons and limes, are highly similar.

### 5.6 Specular reflection

When light hits the surface of a glossy object, two physically distinct reflections occur: diffuse and specular. The spectral distribution of diffuse reflection, as explained in Fig. 2, is determined by the spectral reflectance of the object and the spectral distribution of the illuminant. For a matte surface without gloss, the reflected light depends solely on this diffuse reflection. Specular reflection, however, directly reflects the illuminant without altering its spectral distribution. The final reflected light from a glossy object is the weighted sum of the diffuse and specular reflections, making the illuminant color not directly discernible. However, studies have shown that specular reflection can aid in achieving color constancy [29,30].

### 5.7 Invariant cone-response ratio

Natural lighting and objects typically have broad-band spectral shapes. Within these constraints, the ratio of the L-, M-, and S-cone response of any two surfaces remains constant despite changes in scene illuminant [31]. Thus, a change in the cone-response ratio suggests a change in object color, not lighting. Although this does not determine the absolute illuminant color, it is proposed as a strategy to distinguish whether a perceived color change is due to changes in illuminant or reflectance. This strategy may be used by the visual system [31,32].

## 6 Conclusion

This tutorial review discussed challenges associated with color constancy, highlighted regularity in the natural world and its effect on the sensory signals, and visual cues for estimating illuminant color. Although psychophysical experiments were not throughly covered, they are crucial for understanding color constancy. Since the first measurements in 1986 [33], various experiments have revealed the extent and conditions under which human color constancy holds. For further reading, refer to review articles [34,35]. Recent advances include illuminant-estimation models using complex algorithms [36] and a color-constancy model using deep learning [37]. However, the physiological basis of color constancy, particularly processing in the cerebral cortex, is yet fully understood [38–40]. Future studies are expected to address this.

Perceptual constancy transforms unstable physical signals into a stable perceptual world, supporting our robust behaviour. Understanding this mechanism will enhance our knowledge of brain function. Moreover, insights into perceptual constancy have been applied to various technologies. White-balance adjustment, for instance, is used in devices such as smartphones to naturally corrects the color of images captured under different illuminants. In computer vision, constancy enables safer technologies, such as object-recognition systems in autonomous vehicles that must identify objects and humans to avoid, regardless of surrounding environmental conditions. In the textile and paint industries, it aids in designing spectral reflectance to ensure consistent color appearance despite changes in illuminants. Additionally, understanding color constancy addresses issues such as online clothing appearing different under home lighting and ensures accurate artistic impressions in museums. Moving forward, integrating perspectives from various fields such as physics, computer science, psychology, and physiology may further elucidate the mechanism of color constancy.
